# Magnetic Resonance-guided External Beam Radiation and Brachytherapy for a Patient with Intact Cervical Cancer

**DOI:** 10.7759/cureus.2577

**Published:** 2018-05-04

**Authors:** David Asher, Kyle R Padgett, Ricardo E Llorente, Benjamin S Farnia, John C Ford, Shefali R Gajjar, Shahil Mehta, Garrett N Simpson, Nesrin Dogan, Lorraine Portelance

**Affiliations:** 1 Department of Radiation Oncology, Sylvester Comprehensive Cancer Center, Miller School of Medicine, University of Miami, Miami, USA

**Keywords:** cervical cancer, mr guided radiotherapy, mr-guided brachytherapy

## Abstract

Radiation treatment verification has improved significantly over the past decades. The field has moved from film X-rays and skin marks to fiducial tracking and daily cone beam computed tomography (CBCT) for tumor localization. We now have the ability to perform daily on-board magnetic resonance imaging (MRI), which provides superior soft tissue contrast compared to computed tomography (CT). In the management of cervical cancer, the brachytherapy literature has demonstrated that MRI allows for better delineation of the high-risk clinical target volume (HR-CTV) and the use of MRI-guided brachytherapy has translated into improved treatment outcomes.

Consensus contouring guidelines for intensity modulated radiation therapy (IMRT) for cervical cancer advise including the whole uterus in the target volume and adding large planning target volume (PTV) margins to account for inter-fractional uterine motion and target motion resulting from variable rectal and bladder filling. MRI-guided radiation therapy (MRgRT) systems enable the possibility to precisely delineate the target volume on a daily basis and to perform truly adaptive delivery. This advancement in technology provides the opportunity to explore how external beam treatment volumes could be safely reduced for better sparing of pelvic organs for the benefit of our patients with cervical cancer.

We describe the MR-guided definitive external beam radiation therapy and brachytherapy for a 32-year-old woman with intact cervical cancer. We contoured the uterus, bladder, rectum, and gross tumor volume (GTV) on each of her 25 set-up MRIs. We demonstrate a steady reduction in the GTV and increased displacement of the uterus and GTV as the GTV decreased in size. The findings presented suggest that cervical cancer could greatly benefit from an adaptive MRgRT approach.

## Introduction

MRI-guided brachytherapy (MRgBT) for intact cervix has been shown to decrease treatment toxicity compared to point-based brachytherapy [1]. In addition, for patients with more advanced disease, MRgBT provides better tumor control [2]. There is evidence in the brachytherapy literature that delineation of the high-risk clinical target volume (HR-CTV) on a computed tomography (CT) overestimates the actual volume. Thus MRI-guided target definition is considered superior especially for tumors extending to the parametrial tissue [3].

With the advent of MRI-guided radiation therapy (MRgRT), tumor and organs at risk (OARs) visualization has markedly improved and truly daily adaptive delivery is made possible. These developments in technology might bring an opportunity to reconsider how target volumes are defined for cervical cancer. Consensus guidelines for MRI-guided contouring of cervical cancer recommend treatment of entire uterus with large margin for target motion [4].

The use of diagnostic pelvic MRI for initial workup has been adopted as standard of care in most developed countries. By utilizing diagnostic MRI, radiation oncologists can precisely contour the gross tumor volume (GTV) by performing a fusion of the MRI with the planning CT. Surgical series have demonstrated that isolated recurrences within the uterine fundus are rare, approximately 2% [5]. Nevertheless, the contouring consensus guidelines recommend to include the fundus in the target volume. This recommendation was justified by the fact that when these guidelines were written, diagnostic MRIs were not widely utilized and that daily treatment verification was based on cone beam computed tomography (CBCT), which does not allow for good tumor visualization [6]. Prior vector analysis using daily setup imaging has demonstrated that relative to the mid-uterus and cervix, the uterine fundus is most likely to be underdosed due to organ motion. Therefore when the intent is to treat the whole uterus a large planning target volume (PTV) is required to ensure adequate dose coverage [7]. Compounding this issue is the substantial inter-fractional target motion observed for cervical-cancer patients [8]. This translates in a need to include a large internal target volume (ITV) margin in the PTV to account for this inter-fractional motion throughout the course of treatment. MRgRT systems offer the possibility to perform on-board adaptive treatment when needed, hence decrease the need for inter-fractional ITV.

The potential benefit of intensity-modulated radiation therapy (IMRT) via OAR sparing is decreased when the entire fundus is included in the clinical target volume (CTV) and a large PTV margin is added to account for fundus motion and OARs volume changes [8]. MRI has superior soft tissue delineation compared to CT, and thus we hypothesize that the PTV could be decreased with the use of MRgRT. With this case study, we present a comprehensive approach where MRI guidance was used during the entire treatment course, from the first day of external beam pelvic radiation to treatment completion with brachytherapy, and we propose that the benefit of adaptive MRgRT should be further explored.

## Case presentation

A 32-year-old pre-menopausal woman presented after a routine pap smear revealed a high-grade squamous intraepithelial lesion (HSIL). Subsequent cold knife cone revealed an infiltrating poorly differentiated squamous cell carcinoma with lymphovascular invasion. A pelvic MRI with and without contrast showed a non-enhancing cervical lesion, 3.5 x 3.2 x 4.6 cm in size. The mass was T1 isointense and T2 heterogeneously intense with evidence of left parametrial extension that was palpable on bimanual exam. MRI and positron emission tomography (PET)/CT revealed no pelvic lymphadenopathy or definitive metastatic disease. She was staged as FIGO IIB and recommended to undergo concurrent chemoradiation, but ultimately was unable to receive chemotherapy due to an atypical mycobacteria pulmonary infection. She received 45 Gray (Gy) in 25 fractions to the PTV, which encompassed the GTV, cervix, parametrium, uterus, upper vagina and pelvic lymph nodes. The external bean treatment was delivered using IMRT on a Tri-Cobalt-60 MRI-guided radiotherapy system (ViewRay MRIdian, Cleveland, Ohio, United States). She was advised to have comfortable bladder filling for treatment. She tolerated external beam radiation with moderate fatigue endorsed toward the end of her treatment course and without gastrointestinal, genitourinary, or integumentary toxicities.

**Figure 1:**
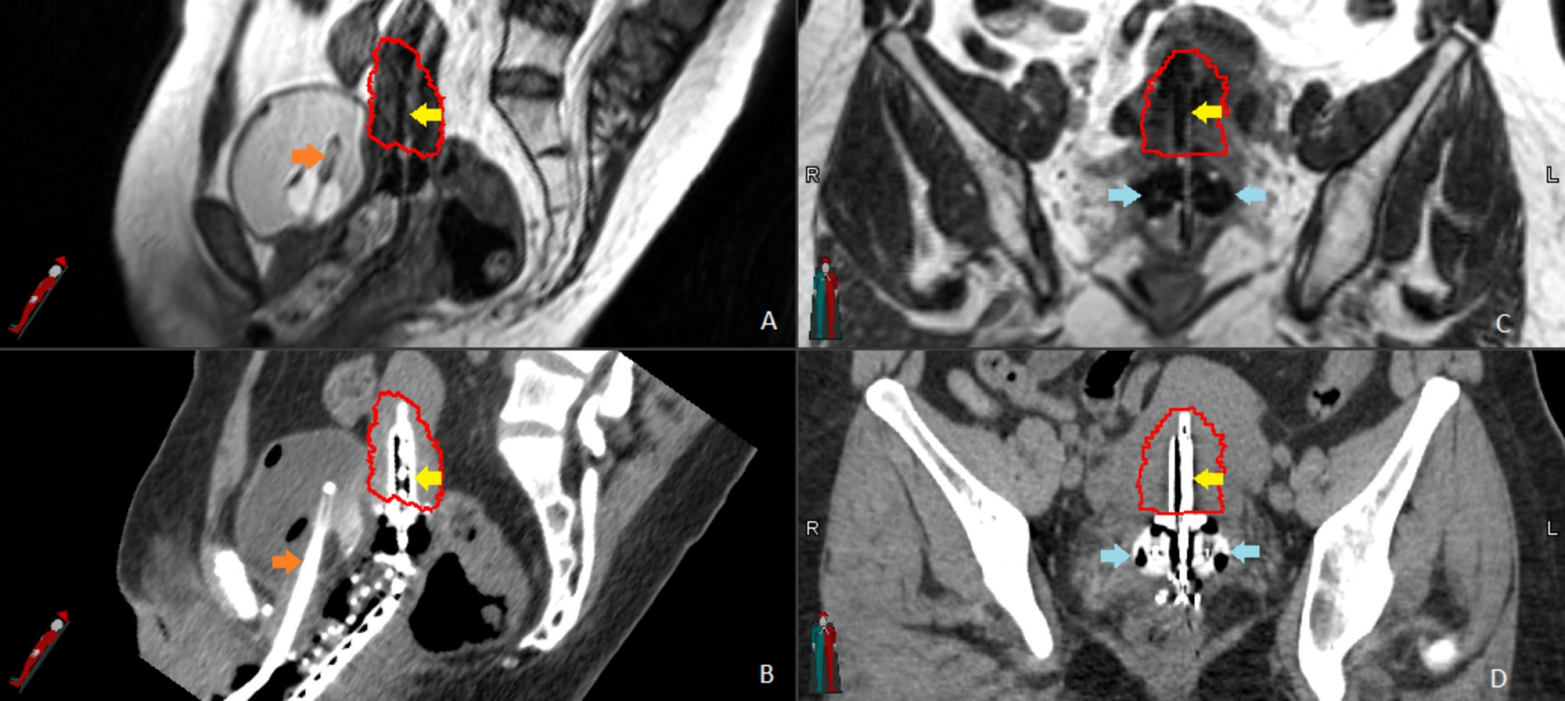
Magnetic resonance-guided brachytherapy. (A) and (B) show the uterine tandem (yellow arrows) and foley catheter (orange arrows) in the sagittal plan on the magnetic resonance imaging (MRI) and computed tomography (CT). The high-risk clinical target volume (HR-CTV) is outlined in red. (C) and (D) show HR-CTV, tandem (yellow arrows), and ovoids (blue arrows) in the coronal plane, again on the respective MRI and CT.

She went on to receive high-dose-rate (HDR) brachytherapy with four intracavitary implants. On the day of the first implant, a Smit sleeve was inserted to facilitate subsequent tandem insertion. An MR-compatible, intra-uterine tandem and two 25 mm ovoids were used for each implant. Ultrasound was utilized intraoperatively to assure proper placement of the applicators. A CT scan was performed for treatment planning which was followed by an MRI study on the MRIdian system for tumor visualization (Figure 1). The CT and MRI were then registered and transferred to the MIM (MIM Software Inc., Cleveland, Ohio, United States) for target delineation. After contouring, the structure set was transferred to Oncentra (Elekta, Stockholm, Sweden) for treatment planning. For each implant, a dose of 7 Gy was prescribed to ensure 90% coverage of the HR-CTV. The patient tolerated brachytherapy without acute side effects. As of her last follow-up, 12 months after completion of radiation, she remains without evidence of disease and the only treatment-related side effect is early menopause.

**Figure 2:**
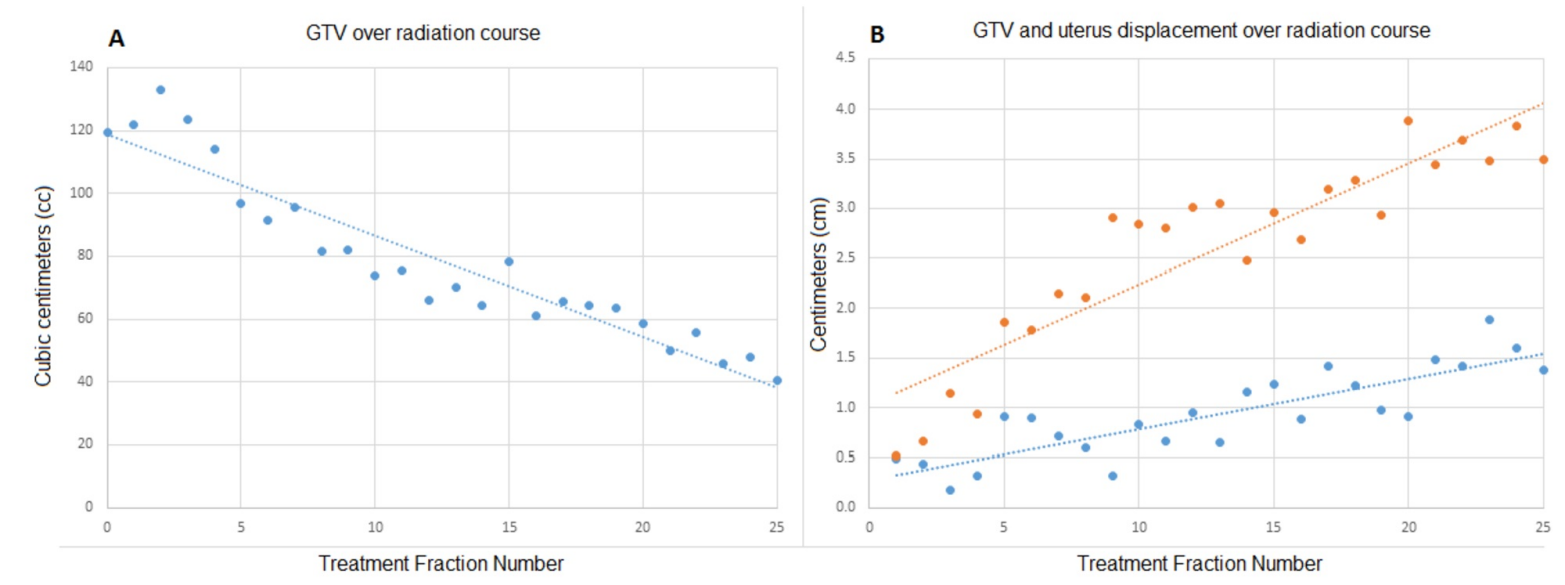
Gross tumor volume (GTV) and displacement. (A) demonstrates the GTV (blue) for each fraction of external beam radiation therapy (RT). (B) shows the displacement of the GTV (blue) and uterus (orange) also for each daily fraction.

Under an institutional review board (IRB)-approved protocol, each of the patient’s 25 daily setup MRIs were retrospectively contoured to include the GTV, bladder, uterus, and rectum. The daily MRIs were registered with the planning MRI to assess organ motion. GTV motion was assessed via GTV displacements. GTV and organ volumes along with GTV and uterus displacements were determined in order to evaluate the relationship between GTV motion and bladder and rectal volumes. As seen in Figure 2, the GTV volume dramatically decreased throughout the course of treatment. In parallel, the amount of GTV displacement increased throughout the treatment course and was most apparent in the caudal direction. In this case, the variation of bladder and rectal filling did not correlate strongly with GTV and uterus displacement.

## Discussion

To the best of our knowledge, this is the first published report of a patient with cervical cancer treated on an MRgRT system. Squamous cell carcinoma of the cervix is generally considered a radiosensitive tumor. In the clinical case presented here, despite the fact that this patient could not receive chemotherapy, steady regression of the GTV was demonstrated during the treatment (Figure 2). In parallel to the observed tumor shrinkage, GTV displacement increased during the treatment course, indicating that as the GTV decreases in size its mobility is increased.

**Figure 3:**
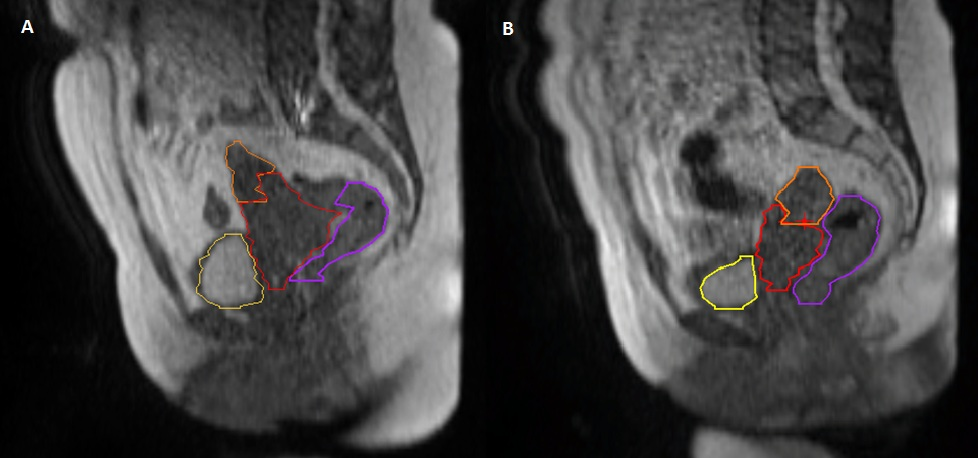
Daily set-up magnetic resonance imaging (MRI) for fraction 1 and 25. Daily set-up imaging using an on-board 0.35-Tesla MRI scanner and a balanced steady state free precession sequence in the sagittal plane for (A) fraction 1 and (B) fraction 25. The bladder (yellow), rectum (purple), gross tumor volume (GTV) (red), and uterus (orange) were contoured on each daily setup MRI. Note the significant reduction in the GTV size, as well as the change in uterine position reflecting inter-fractional motion.

In this case, rectal and bladder volumes did not significantly impact GTV displacement, but in our institutional experience, differences in rectal and bladder filling have been shown to lead to GTV displacement in other patients. Interestingly, the magnitude of uterine displacement also increased during the course of treatment and to a greater degree than the GTV itself (Figure 2). This is concordant with prior vector-based data, which demonstrated increased motion of the fundus relative to the cervix as treatment progressed [7]. We suspect that the initial bulky tumor restrained uterus movement and as the tumor decreased in size, the uterus became less restricted (Figure 3). Figure 3 demonstrates tumor regression from fraction 1 to fraction 25, as well as the large inter-fractional motion of the uterus. If the goal of including the entire uterus within the CTV was maintained, a large PTV would continue to be needed. However, we believe daily MRgRT could allow for a safe transition to a primary target volume limited to the GTV, parametrium, and pelvic lymph nodes with an intra-uterine and a vaginal CTV margin rather than a large extension to cover the whole uterus.

This MRgRT system also demonstrated significant versatility as we were able to use the unit for MR-based brachytherapy.

We now have the ability to utilize adaptive MRgRT to maximally spare organs at risk during the course of pelvic external beam radiation and combine it with MRI-guided adaptive dose escalation brachytherapy. Typically the dose delivered during a course of external beam pelvic radiation for these patients will be in the range of 45-50.4 Gy in 25-28 fractions (with or without a boost to positive pelvic nodes, depending on institution policy). This dose is within the tolerance of the surroundings OARs. However, when considering the potential benefit of adaptive MRgRT for this patient population, one has to keep in mind that just slightly more than half of the total dose required to cure these tumors is being delivered during external beam, the guidelines recommend to deliver a total dose of 85 Gy to the HR-CTV to achieve tumor control [9]. Composites obtained from external beam and image-guided brachytherapy clearly demonstrate that the OARs (bladder, rectum, sigmoid) receive a very high total dose and therefore any advances that could safely decrease the dose to OARs might bring meaningful clinical benefit for patients with cervical cancer.

## Conclusions

This case presentation reflects the dynamic nature of advancements in radiation oncology. While the findings in this report should be followed by additional studies before reproducible conclusions can be drawn, we postulate that MRgRT will be increasingly utilized, and subsequently will bring changes to the way target volumes are defined for external beam radiation. The potential for adaptive radiation therapy is particularly relevant for this tumor site, and its implementation could lead to decreased treatment toxicity.
